# Bleeding-related outcomes after transurethral resection of prostate in patients with chronic antiplatelet or anticoagulant therapy following perioperative interruption: a retrospective cohort study

**DOI:** 10.1186/s12894-026-02220-3

**Published:** 2026-06-09

**Authors:** Abdullah Ilktac, Bayram Dogan, Cevper Ersoz, Senad Kalkan, Serkan Akıncı, Muzaffer Akcay, Yusuf Ozlem Ilbey

**Affiliations:** 1 Faculty of Medicine, Department of Urology, Bezmialem Vakif University, Adnan Menderes Bulvarı, Vatan Caddesi, Fatih, Istanbul, 34093 Turkey; 2 Faculty of Medicine, Department of Urology, Istanbul Medipol University, Şht. Emin Colen Sokağı No:4, Kadikoy, Istanbul, 34718 Turkey; 3 Pelvic Urology Centre, Teşvikiye, Hakkı Yeten Cd. Doğu İş Merkezi No:15/10 kat 4, Şişli, Istanbul, 34365 Turkey

**Keywords:** Benign prostatic hyperplasia, TURP, Antiplatelet therapy, Anticoagulant therapy, Bleeding complications, Hematuria

## Abstract

**Background:**

Transurethral resection of the prostate (TURP) is s the gold standard surgical treatment method for benign prostatic hyperplasia. With the aging population and the increasing prevalence of cardiovascular diseases, the number of patients requiring anticoagulant (AC) or antiplatelet (AP) therapy continues to rise. The perioperative management of these medications remains challenging because continuation may increase bleeding complications, whereas discontinuation may increase thromboembolic risk. In this study, we evaluated bleeding-related outcomes after TURP in patients receiving chronic AP or AC therapy undergoing perioperative medication interruption.

**Methods:**

This retrospective study included patients who underwent TURP between January 2020 and December 2024 at our tertiary referral center. Patients were divided into three groups according to their perioperative use of AP and AC medications. Control group: Patients who did not receive any AC or AP therapy. AP group: Patients receiving antiplatelet therapy. AC group: Patients treated with anticoagulants. None of the patients underwent TURP under continued AP or AC therapy. Perioperative and postoperative outcomes, including duration of irrigation, duration of catheterization, length of hospital stay, development of clot retention during hospitalization, need for postoperative blood transfusion, readmission due to hematuria within 30 days, and need for recatheterization or reoperation due to bleeding, were evaluated. A composite bleeding outcome was created to increase statistical power including postoperative blood transfusion, clot retention, and hematuria-related readmission.

**Results:**

A total of 325 patients were included: 198 in the control group, 97 in the AP group, and 30 in the AC group. Overall comparisons showed significant differences in postoperative transfusion (*p* = 0.001) and hematuria-related readmission rates (*p* = 0.032) between three groups but post-hoc pairwise Fisher’s exact tests with Bonferroni correction did not confirm statistical significance between specific pairs. There were no significant differences between the groups in other parameters. Multivariable logistic regression identified antiplatelet therapy as an independent predictor of hematuria-related readmission (OR:4.17, *p* = 0.010).

**Conclusion:**

AP therapy is independently associated with an increased risk of hematuria-related readmission even after perioperative interruption of therapy. Patients receiving antithrombotic therapy should be carefully monitored during the perioperative period, and clinicians should remain vigilant for potential bleeding-related complications.

## Background

Transurethral resection of the prostate (TURP) is still accepted as the gold standard surgical treatment method for patients with bothersome moderate to severe lower urinary tract symptoms (LUTS) due to benign prostatic hyperplasia (BPH) [1]. Although several minimally invasive and laser-based alternatives have been developed, TURP remains widely performed because of its proven efficacy, durable long-term outcomes, and economic feasibility. The prevalence of moderate-to-severe LUTS rises progressively with age, reaching over 40% among men in their seventh and eighth decades [2, 3]. Consequently, the rate of surgical intervention for BPH also increases among men in older age groups. TURP is a high bleeding-risk procedure with an estimated 30-day risk of major bleeding greater than 2% [4, 5]. Blood transfusion and clot retention rates after bipolar TURP were reported as 2% and 5%, respectively [6, 7].

With the aging population and the increasing prevalence of cardiovascular diseases, the number of patients requiring anticoagulant (AC) or antiplatelet (AP) therapy continues to rise [8, 9]. This creates a significant perioperative challenge, as continuation of these medications may further increase the prevalence of bleeding-related complications after TURP, whereas their discontinuation may lead to potentially serious thromboembolic events. Laser-based techniques such as holmium laser enucleation of the prostate (HOLEP) have been reported to be safely performed in patients using antithrombotic therapy, with comparable complication rates [10]. However, these technologies are not universally available and are associated with higher costs; therefore, TURP remains the most widely performed surgical option for many patients.

The present study aimed to evaluate perioperative and postoperative bleeding-related outcomes after TURP in patients with chronic AP or AC therapy following perioperative interruption, with particular emphasis on the separate analysis of AP and AC cohorts and factors associated with hematuria-related readmission.

## Materials and methods

### Study design and patient selection

This is a retrospective study evaluating prospectively collected data of the patients who underwent TURP between January 2020 and December 2024 at our tertiary referral center. Patients were divided into three groups according to their perioperative use of AP and AC medications. Control group: Patients who did not receive any AC or AP therapy; AP group: Patients receiving antiplatelet therapy and AC group: Patients treated with warfarin or direct oral anticoagulants. Patients with incomplete medical records, a history of previous TURP, combined bladder tumor resection, known prostate cancer or concomitant upper urinary tract surgery were excluded from the study. Also, patients receiving dual antithrombotic therapy were excluded from the study cohort to avoid potential confounding effects on bleeding outcomes and to allow a clearer comparison between the study groups.

### Perioperative management

All procedures were performed using a standard bipolar resection system with isotonic saline irrigation. In our institution, the decision regarding perioperative management of AP and AC therapy, including timing of drug discontinuation and the use of low molecular weight heparin (LMWH) bridging therapy, was determined through multidisciplinary discussion involving the anesthesiology team, the treating physician who initiated the antithrombotic treatment, and the operating urologist according to the patient’s underlying medical condition and thromboembolic risk profile. Decisions regarding interruption, bridging, and perioperative management were primarily guided by the recommendations of the anesthesiology team and the physician who initiated the antithrombotic therapy. The operating urologist was mainly responsible for postoperative bleeding assessment and determination of the appropriate timing for discontinuation of LMWH and resumption of the patient’s original antithrombotic therapy. AP therapy was discontinued 5–7 days before surgery. In the AC group, warfarin therapy was discontinued 5 days before surgery and direct oral anticoagulants were interrupted 3 days preoperatively. LMWH bridging was considered in patients at high thromboembolic risk and the dosing was made by the anesthesiology team or the physician responsible for the patient’s antithrombotic therapy. Antithrombotic therapy was restarted postoperatively after adequate hemostasis was achieved. None of the patients underwent TURP while receiving AP or AC therapy. If interruption of AC or AP therapy was contraindicated, patients were referred to other centers for alternative treatment approaches. All patients received bladder irrigation postoperatively, and continuous irrigation was maintained until the effluent became macroscopically clear.

### Data collection

The following preoperative variables were recorded: age, body mass index (BMI), preoperative hemoglobin level, international normalized ratio (INR), prostate volume, International Prostate Symptom Score (IPSS), and quality-of-life score.

Perioperative and postoperative outcomes included: Duration of irrigation, duration of catheterization, length of hospital stay, development of clot retention during hospitalization, need for postoperative blood transfusion, readmission due to hematuria within 30 days, and need for recatheterization or reoperation due to clot retention. Patients who developed postoperative clot retention were initially managed conservatively with catheter irrigation and/or catheter evacuation. Patients who did not respond to conservative measures underwent surgical management with endoscopic clot evacuation. Hematuria-related readmission was defined as hospital readmission within 30 days due to gross hematuria requiring clinical evaluation and management, including catheterization, bladder irrigation, transfusion, or intervention. Postoperative transfusion decisions were made according to institutional clinical practice based on hemoglobin level, hemodynamic status, ongoing bleeding, and clinician judgment. As individual bleeding-related events were relatively infrequent, a composite bleeding outcome was created to increase statistical power. The composite outcome was defined as the occurrence of at least one of the following events: postoperative blood transfusion, clot retention, or hematuria-related readmission.

### Statistical analysis

Statistical analyses were performed using NCSS (Number Cruncher Statistical System) 2007 (Kaysville, Utah, USA). Data distribution was assessed using the Shapiro–Wilk test. Variables following normal distribution were expressed as mean ± standard deviation (SD) and compared using one-way ANOVA, with Bonferroni-adjusted post-hoc t-tests for pairwise comparisons. Non-normally distributed continuous variables were expressed as median (interquartile range, IQR) and compared using the Kruskal–Wallis test. Categorical variables were compared using Fisher’s Exact test, and significant results were further analyzed with Bonferroni-adjusted pairwise Fisher’s tests. A p-value < 0.05 was considered statistically significant.

## Results

### Patient characteristics

A total of 325 patients who underwent TURP were included: 198 in the control group (no AP or AC therapy), 97 in the AP group, and 30 in the AC group. In the AP group, 74 (76.3%) patients were using clopidogrel, and 23 (23.7%) were using acetylsalicylic acid. In the AC group, 17 (56.6%) patients were using rivaroxaban, 8 (26.7%) were using apixaban, and 5 (16.7%) were using coumadin. Age differed significantly among the three groups (*p* < 0.001), with the anticoagulant group being the oldest (74.8 ± 7.0 years), followed by the antiplatelet group (70.7 ± 6.9 years) and the control group (66.5 ± 8.1 years). Post hoc pairwise comparisons with Bonferroni correction confirmed significant differences among all groups (Control vs. AC: *p* < 0.001; Control vs. AP: *p* < 0.001; AC vs. AP: *p* = 0.005). No significant differences were observed between the groups in terms of BMI, INR, preoperative hemoglobin, prostate volume, IPPS, or quality-of-life score values (Table 1).

**Table 1:** Preoperative demographic and clinical characteristics of the study groups

	TURP with AP (*n* = 97)	TURP with AC (*n* = 30)	Control (*n* = 198)	*p*
Age, years	70.7 ± 6.9	74.8 ± 7.0	66.5 ± 8.1	< 0.001
BMI	27.9 ± 4.5	27.8 ± 3.4	28.3 ± 5.9	0.314
INR	1.05 ± 0.10	1.06 ± 0.12	1.03 ± 0.08	0.643
Hemoglobin	13.67 ± 1.56	13.27 ± 1.21	13.98 ± 1.61	0.729
Prostate volume, ml	72.0 ± 34.5	73.0 ± 22.8	72.3 ± 29.5	0.726
IPSS	23.9 ± 6.6	22.0 ± 6.4	22.6 ± 6.0	0.077
Quality of Life	4.4 ± 1.0	4.4 ± 1.0	4.4 ± 1.0	0.995

*TURP* Transurethral resection of prostate, *AP* Antiplatelet therapy, *AC* Anticoagulant therapy, *BMI* Body mass index, *IPSS* International Prostate Symptom Score

### Perioperative and postoperative outcomes

The mean time to restart antithrombotic therapy was similar between the antiplatelet and anticoagulant groups (3.38 ± 1.25 vs. 3.23 ± 1.41 days, respectively), with no statistically significant difference between the groups (*p* = 0.624). Bridging with LMWH was performed in 21 (21.6%) patients in the AP group and in 18 (60%) patients in the AC group. Overall, there were no significant differences among the groups regarding median duration of irrigation (*p* = 0.272), duration of catheterization (*p* = 0.148), or length of hospital stay (*p* = 0.152). Similarly, the rates of in-hospital clot retention (*p* = 0.421), clot retention after discharge requiring evacuation with catheter (*p* = 0.135), or cystoscopic evacuation (*p* = 0.779) were comparable across groups. However, significant overall differences were observed in postoperative transfusion (*p* = 0.001) and readmission due to hematuria (*p* = 0.032) (Table 2). Post hoc pairwise Fisher’s exact tests with Bonferroni correction did not demonstrate statistically significant differences between the individual groups. For postoperative transfusion, pairwise p-values were 0.329 (Control vs. AP), 0.017 (Control vs. AC), and 0.139 (AP vs. AC), all adjusted *p* > 0.017. For readmission, pairwise p-values were 0.017 (Control vs. AP), 0.336 (Control vs. AC), and 0.732 (AP vs. AC), again, all adjusted *p* > 0.017. Although the overall comparisons suggested increased bleeding-related complications in patients using antithrombotic therapy, these differences did not remain statistically significant after correction for multiple testing. Composite bleeding outcome occurred in 3.5% of patients in the control group, 12.4% in the antiplatelet group, and 13.3% in the anticoagulant group. Overall comparison showed a significant difference among groups (*p* = 0.008). Pairwise analyses demonstrated a higher risk in the AP group compared with the control group (*p* = 0.009), while other comparisons were not statistically significant after correction for multiple testing (Table 3). In patients with hematuria-related readmission, the mean interval between catheter removal and readmission was 14.6 ± 9.4 days in the control group, 10.1 ± 8.1 days in the AP group, and 5.0 ± 0 days in the AC group.

**Table 2:** Perioperative and postoperative bleeding-related outcomes after TURP according to antiplatelet and anticoagulant therapy status

	TURP with AP (*n* = 97)	TURP with AC (*n* = 30)	Control (*n* = 198)	*p*
Duration of irrigation, median (IQR), days	1 (1–1)	1 (1–1)	1(1–1)	0.272
Hospital stay, median (IQR), days	1 (1–1)	1 (1–1)	1 (1–1)	0.152
Duration of catheterization median (IQR), days	4 (3–5)	5 (3–6)	5 ( 4–6)	0.148
Clot retention during hospitalization, n (%)	6 (6.2)	1 (3.3)	6 (3)	0.421
Patients with postop. transfusion, n (%)	1 (1)	2 (6.7)	0	**0.001**
Readmission with hematuria, n (%)	11 (11.6)	2 (6.7)	7 (3.5)	**0.032**
Clot retention requiring evacuation with catheter, n (%)	8 (8.2)	2 (6.7)	6 (3)	0.135
Clot retention requiring cystoscopy, n (%)	1 (1.0)	0 (0)	1 (0.5)	0.779

*TURP* Transurethral resection of prostate, *AP* Antiplatelet therapy, *AC* Anticoagulant therapy, *Postop*. Postoperative

**Table 3:** Composite bleeding outcome according to antiplatelet and anticoagulant therapy

	TURP with AP (*n* = 97)	TURP with AC (*n* = 30)	Control (*n* = 198)	p	Pairwise comparisons (Bonferroni corrected)
Composite bleeding outcome, n (%)	12 (12.4%)	4 (13.3%)	7 (3.5%)	0.008	Control vs. AP: **0.009**; Control vs. AC: 0.042; AP vs. AC: 1.000

*AP* Antiplatelet therapy, *AC* Anticoagulant therapy. Composite bleeding outcome: Postoperative transfusion, clot retention, or hematuria-related readmission. Overall comparison performed using the chi-square test. Pairwise comparisons were performed using Fisher’s exact test with Bonferroni correction (significance threshold *p* < 0.017)

### Multivariable logistic regression analysis

Multivariable logistic regression identified AP use as the only independent predictor of hematuria-related readmission after TURP (OR = 4.17, *p* = 0.010). Patients on antiplatelet therapy had nearly four-fold higher odds of repeat hospital visits compared to the control group. Anticoagulant use was associated with higher, though non-significant, odds of readmission (OR = 3.53, *p* = 0.193). Neither age (*p* = 0.122), BMI (*p* = 0.833), prostate volume (*p* = 0.571), IPSS (*p* = 0.421), nor LMWH bridging (*p* = 0.651) significantly influenced readmission risk (Table 4).

**Table 4:** Multivariable logistic regression analysis for hematuria-related readmission after TURP

Variable	Odds Ratio (OR)	95% Confidence Interval	*p*
Age, years	0.95	0.90–1.01	0.122
Body mass index	1.00	0.96–1.05	0.833
Prostate volume, ml	1.00	0.99–1.02	0.571
IPSS	1.03	0.96–1.11	0.421
Antiplatelet therapy	4.17	1.40–12.42	**0.010**
Anticoagulant therapy	3.53	0.53–23.54	0.193
LMWH Bridging	0.72	0.17–3.01	0.651

*OR* Odds ratio, *CI* Confidence interval, *BMI* Body mass index, *IPSS* International Prostate Symptom Score, *LMWH* Low molecular weight heparin

## Discussion

In this retrospective study, we evaluated the impact of perioperative AC and AP therapy on bleeding-related complications following TURP. Overall group comparisons demonstrated significant differences in postoperative transfusion and hematuria-related readmission rates. However, post hoc pairwise comparisons with Bonferroni correction showed no statistically significant differences between individual groups. This discrepancy may be explained by the relatively small sample sizes within subgroups. To overcome this limitation, a composite bleeding outcome including postoperative transfusion, clot retention, and hematuria-related readmission was analyzed and we found that the bleeding-related complication rate was significantly higher in the AP group compared with the control group. Importantly, multivariable logistic regression analysis identified AP therapy as an independent predictor of hematuria-related readmission after TURP. These findings suggest a clinically relevant association between AP therapy and bleeding-related complications.

The available literature on the impact of antithrombotic therapy on bleeding outcomes after TURP remains heterogeneous and sometimes contradictory. Some studies have reported an increased bleeding risk in patients receiving AP therapy. In contrast, others have demonstrated higher complication rates in patients treated with anticoagulants or those undergoing perioperative LMWH bridging. Perioperative management strategies vary substantially among studies. In some reports, patients continued antithrombotic therapy during surgery, while in others, medications were temporarily discontinued before the procedure. This variability in patient selection, antithrombotic regimens, and perioperative management strategies makes direct comparison between studies challenging and may partly explain the inconsistent findings reported in the literature. Kuo et al. retrospectively evaluated 629 patients who underwent TURP, of whom 113 (17.9%) were using AC medication [11]. All patients in the anticoagulant group discontinued their medication preoperatively. Bridging with LMWH was performed in 21.2% of the patients in the AC group. They reported that the AC group had significantly higher rates of transfusion, hematuria-related readmission, and prolonged hematuria than the non-AC group. In the same cohort, there were 166 patients receiving AP medication and these patients were included in the Non-AC group. Multivariable analyses demonstrated that antiplatelet therapy was an independent predictor of bleeding-related readmission following TURP. Ong et al. evaluated perioperative antithrombotic management in 293 patients undergoing TURP [12]. Among these patients, 107 (37%) were receiving AP therapy, and 25 (9%) were receiving AC therapy. In the AP group 35 (33%) patients continued AP therapy during the perioperative period and 72 (67%) patients discontinued preoperatively. All patients in the AC group discontinued their medication before the operation and bridging with LMWH was performed in 18 (72%) patients. Postoperative bleeding-related complications like prolonged irrigation, clot retention, transfusion and reoperation occurred in 10% of the cases. AC users who received LMWH bridging had significantly higher bleeding-related complications compared to other groups. Li et al. evaluated perioperative outcomes in 31 patients with a history of antithrombotic therapy undergoing TURP [13]. The majority of patients were receiving antiplatelet therapy, including aspirin (61.3%), clopidogrel (19.4%), or dual antiplatelet therapy (12.9%), while only two patients (6.5%) were treated with warfarin. After temporary discontinuation of antithrombotic medication before surgery and without perioperative bridging therapy, no patients required blood transfusion or surgical intervention for bleeding, and only one patient required additional bladder irrigation due to clot obstruction. In our cohort, although overall group comparisons suggested significant differences in postoperative transfusion and hematuria-related readmission rates, pairwise comparisons between the AP, AC, and control groups did not remain statistically significant after Bonferroni correction. Multivariable analysis revealed that antiplatelet therapy was the only independent predictor of hematuria-related readmission after TURP. No significant association was observed between LMWH bridging therapy and hematuria-related readmission in our study population. Taken together, these findings highlight the heterogeneity of the current literature regarding the relative impact of AP therapy, AC use, and LMWH bridging on bleeding-related outcomes after TURP. A recent meta-analysis evaluating the perioperative use of AC and AP medications in BPH surgery demonstrated that the continuous use of these agents significantly increased postoperative bleeding and transfusion rates after TURP [14]. In this analysis, the risk of postoperative bleeding was more than four times higher in patients who continued therapy compared with those who discontinued treatment before surgery (OR = 4.34), and the risk of blood transfusion was also significantly increased (OR = 2.96). These findings support the notion that perioperative AP and AC therapy may contribute to increased bleeding complications following TURP. When compared with patients not receiving any AC or AP therapy, continuous use of these agents was associated with an even greater bleeding risk (OR = 5.52). The authors also emphasized that perioperative management strategies varied substantially across studies, and the role of LMWH substitution during temporary discontinuation remains controversial. In another similar meta-analysis, Liang et al. reported that patients on AC/AP therapy had higher bleeding-related complications and transfusion rates after TURP compared to the control group [15]. HOLEP has been increasingly preferred in patients receiving antithrombotic therapy due to its superior hemostatic properties. Pastore et al. 338 patients who underwent HOLEP. Of these patients, 212 (62.7%) received no antithrombotic therapy, 76 (22.5%) received AP, and 50 (14.8%) received AC therapy [16]. They found that patients receiving antithrombotic therapy had longer catheterization and hospital stay, as well as higher rates of readmission and transfusion, while re-intervention rates were similar. AC therapy was identified as an independent predictor of postoperative hemorrhagic complications. In another study, Agarwal et al. evaluated 472 patients undergoing HOLEP, 6.3% were receiving AP therapy and 17.2% AC therapy [17]. They reported that transfusion and clot evacuation rates were comparable between the groups. A higher rate of 90-day postoperative complications was observed in patients receiving AP or AC therapy but no significant difference was found in emergency department visits due to bleeding compared with controls. Although our study did not include patients undergoing HOLEP, the available evidence suggests that similar considerations regarding antithrombotic therapy and bleeding-related outcomes may also apply to these patients.

In our study, AP therapy remained independently associated with hematuria-related readmission despite perioperative interruption, whereas AC therapy did not. Several explanations may account for this finding. First, the smaller size of the AC cohort may have limited statistical power. Second, perioperative management was not identical between the AP and AC groups, including differences in medication interruption and bridging practices. Also, most patients in the AP group were receiving clopidogrel rather than acetylsalicylic acid. Because antiplatelet agents differ in their pharmacologic potency and bleeding risk profiles, the predominance of clopidogrel use in our cohort may have contributed to the observed findings. Different results might have been observed in a cohort predominantly receiving acetylsalicylic acid. These factors may partly explain the different associations observed between AP and AC therapy. Similarly, the composite bleeding outcome remained significantly higher in the AP group compared with controls, despite perioperative interruption of AP therapy. This finding suggests that temporary medication discontinuation alone may not completely eliminate postoperative bleeding risk in this population. Also, postoperative resumption of AP therapy, although clinically necessary, may contribute to delayed postoperative bleeding events in susceptible patients.

Clinically, patients with chronic AP therapy undergoing TURP may benefit from closer postoperative monitoring and counselling regarding delayed hematuria-related complications, even after perioperative interruption of therapy. Postdischarge surveillance may be particularly important in this population because readmissions frequently occurred during the early postoperative period in our cohort.

This study has several limitations. First, it is a retrospective study and the relatively small sample size, particularly in the anticoagulant subgroup, may have limited the statistical power of the analyses. Our study focused exclusively on patients undergoing TURP. Patients treated with newer laser-based surgical techniques such as HOLEP are not included. Finally, we were unable to report the rate of thromboembolic complications, which represent an important clinical outcome, particularly when AP or AC therapy is discontinued during the perioperative period.

## Conclusion

The impact of AP and AC therapy on bleeding-related complications after TURP remains controversial in the current literature. While some studies suggest that AP therapy increases bleeding risk, others report a greater influence of AC therapy or perioperative LMWH bridging. Our findings indicate that AP therapy may be associated with an increased risk of hematuria-related readmission after TURP despite perioperative discontinuation. Therefore, patients receiving antithrombotic therapy should be carefully monitored during the perioperative period. Further prospective studies with standardized antithrombotic protocols, clearly defined medication regimens, and inclusion of contemporary laser-based surgical techniques are needed to better clarify the differential impact of AP therapy, AC therapy, and bridging strategies on postoperative outcomes.

## Data Availability

The datasets generated and/or analysed during the current study are not publicly available due to institutional data protection policies but are available from the corresponding author on reasonable request.

## Abbreviations

TURP: Transurethral resection of the prostate
LUTS: Lower urinary tract symptoms
BPH: Benign prostatic hyperplasia
AC: Anticoagulant
AP: Antiplatelet
HOLEP: Holmium laser enucleation of prostate
LMWH: Low molecular weight bridging therapy
BMI: Body mass index
INR: International normalized ratio
IPSS: International Prostate Symptom Score
